# Implementing PERFECT-ER with Plan-Do-Study-Act on acute orthopaedic hospital wards: Building knowledge from an implementation study using Normalization Process Theory

**DOI:** 10.1371/journal.pone.0279651

**Published:** 2023-02-24

**Authors:** Chris Fox, Simon P. Hammond, Tamara Backhouse, Fiona Poland, Justin Waring, Bridget Penhale, Jane L. Cross

**Affiliations:** 1 Norwich Medical School, Faculty of Medicine and Health Sciences, University of East Anglia, Norwich, England; 2 School of Health Sciences, Faculty of Medicine and Health Sciences, University of East Anglia, Norwich, England; 3 Health Services Management Centre, School of Social Policy, University of Birmingham, Birmingham, England; Sri Eshwar College of Engineering, INDIA

## Abstract

**Background:**

Delivering care to growing numbers of patients with increasingly ‘complex’ needs is currently compromised by a system designed to treat patients within organizational clinical specialties, making this difficult to reconfigure to fit care to needs. Problematic experiences of people with cognitive impairment(s) admitted to hospitals with a hip fracture, exemplify the complex challenges that result if their care is not tailored. This study explored whether a flexible, multicomponent intervention, adapting services to the needs of this patient group, could be implemented in acute hospital settings.

**Methods:**

We used action research with case study design to introduce the intervention using a Plan-Do-Study-Act (PDSA) model to three different hospital sites (cases) across England. The qualitative data for this paper was researcher-generated (notes from observations and teleconference meetings) and change agent-generated (action plans and weekly reflective reports of change agents’ activities). Normalization Process Theory (NPT) was used to analyze and explain the work of interacting actors in implementing and then normalizing (embedding) the intervention across contexts and times. Data analysis was abductive, generating inductive codes then identified with NPT constructs. Across the three cases, change agents had to work through numerous implementation challenges: needing to make sense of the intervention package, the PDSA model as implementation method, and their own role as change agents and to orientate these within their action context (*coherence*). They had to work to encourage colleagues to invest in these changes (*cognitive participation*) and find ways to implement the intervention by mobilising changes (*collective action)*. Finally, they created strategies for clinical routines to continue to self-review, reconfiguring actions and future plans to enable the intervention to be sustained (*reflexive monitoring*).

**Conclusions:**

Successful implementation of the (PERFECT-ER) intervention requires change agents to recognize and engage with local values, and then to enable its fit with practice and wider contextual goals. A context of constant change fragments normalization. Thus, sustaining practice change over time is fragile and requires change agents to continue a recursive two-way sense-making process. This enables implementation and normalization to re-energize and overcome barriers to change.

## Introduction

Care systems are often designed to treat patients within discrete organizational clinical specialties, but this can compromise the delivery of healthcare as integrated and patient-centered, especially for older people with complex health conditions [1–3]. People experiencing cognitive impairment (CI) (including, but not exclusively, dementia and/or delirium) and hip fracture are a notable example, where their treatment creates a complex scenario of multiple simultaneous demands from hospital care [4, 5]. People experiencing CI who break their hip are cared for in environments designed to deliver excellent hip fracture care, but where they may be poorly managed due to a lack of expertise in managing CI. Care of these individuals calls for greater risk management because this group experiences more post-operative complications including delirium and mortality [3, 6–8].

As the demographic of patients change, uptake of relevant research findings into routine healthcare becomes even more vital. Developing and testing interventions for efficacy is important, but so is knowledge of appropriate robust, theory-based implementation to normalize changes [9]. Normalization Process Theory (NPT) is a middle-range conceptual framework that attends to work of implementing, embedding, and sustaining new modes of care into established routines and patterns of social organization [10, 11]. Researchers increasingly use NPT to examine how agents enact change, affected by individual, local, and national contexts, rather than how an intervention should be implemented in ideal circumstances [12–15]. However, an NPT approach can be used to examine changes across different settings and over time with multiple stakeholders [16, 17]. This is important as if evidence-based practice changes are to become part of routine clinical practices, embedding them across different contexts of settings and over time, focusing on sustainability [18].

In this paper we use NPT to understand how implementing, embedding, and integrating a complex intervention, designed to optimize care for patients experiencing CI and hip fracture, was carried out in three National Health Service (NHS) acute orthopedic wards over a twelve-month period [19]. We used the (name removed for peer-review, henceforth PERFECT-ER) programme implementation data to explore the context, procedural dynamics and temporal features of how and to what extent PERFECT-ER became normalized across place and time. We conclude by critically examining what is necessary to normalize healthcare interventions which offer multi-component flexibility, in diverse settings, over time.

## Methods

### Ethics

This study was granted ethical approval by National Research Ethics Service (NRES) Committee South Central—Oxford C (Rec reference number: 15/SC/0294) on 04.06.2015. All participants gave informed consent to participate in this research.

### Aims and design

To assess the acceptability of PERFECT-ER and then to apply knowledge relevant to refine it, we used developmental action research [20–22] with an extended case study design [23–25]. This facilitated implementation data to be collected, for the iterative process of practice change in three acute orthopaedic wards in different NHS hospitals. We treated each ward as a case, using case study methods to assess the practices of each ward and then within- and between-case analysis across sites. The research team observed and interacted with each site as action researchers, collecting observational and documentary data over a 12-month period.

In each site, a Service Improvement Lead (SIL) used the Plan-Do-Study-Act (PDSA) model to lead implementation, for four PDSA cycles over 12 months (each cycle lasting three months) [26]. PDSA provides a model for structuring and encouraging change by recording and reflecting on implementation processes, actively learning about the intervention and its implementation, adjusting, and systematically sharing these lessons across multiple settings and time points [27].

### Settings

This research took place in three English geographical regions selected to embody differences in locations on the rural-urban continuum. Table 1 summarizes information about participating wards.

**Table 1:** SIL and PL characteristics.

Hospital	Ward information	SIL pseudonym	SIL background	PPL pseudonym	PPL background
Site A	32-bed ward in a large urban University Teaching Hospital, admitting 795 hip fractures per year	Elizabeth	Deputy Ward Sister (Band 6); Trauma orthopaedics since 2007	Marcus	Ortho-geriatrician
Site B	28-bed ward in a General Hospital in a suburban area, admitting 524 hip fractures per year	Helen	Ward Staff Nurse since 2007; Elective Orthopaedics and Trauma	Heather	Ortho-geriatrician
Site C	22-bed ward in a small rural University Teaching Hospital with 393 hip fractures per year	Karen	Theatre Nurse since 2005; Trauma Orthopaedics since 2010	Mohamed	Orthopaedic and trauma surgeon

### Description of the intervention

PERFECT-ER is a flexible multicomponent service improvement intervention with an implementation model. The intervention is: the PERFECT-ER checklist (henceforth checklist) which synthesises best practice for hospital-based dementia care with current best practice for hip fracture care, change agents (SIL and PPL) and a model for change (PDSA). Checklists have previously been used in acute care to improve practice [28], however this is not currently in the public domain but described more fully elsewhere [29].

### Change agents: Service Improvement Lead (SIL) & PERFECT-ER Process Leads (PPL)

In each site, trained SILs acted as change agents [30], using the PDSA processes whilst providing critical evaluations of the PERFECT-ER intervention to the study team for refinement. These SILs were nurses, seconded for three days a week from their usual roles. They were supported by a PERFECT-ER Process Lead (PPL) for an hour a week. These were senior hospital-based physicians or surgeons who provided mentoring and liaised with senior medical staff to facilitate implementation (see Table 1 for SIL and PPL demographics). All provided informed consent to participate.

### Participants

NHS staff, including nurses, healthcare assistants (HCAs), physiotherapists and occupational therapists, provide daily care on orthopedic wards. Others, including orthogeriatricians, geriatricians, orthopedic surgeons, anaesthetists, pharmacists, and social workers, spend a proportion of their daily time on individual wards. We recruited any staff who regularly (at least weekly) delivered care on study wards, since they would be involved in delivering care relevant to the PERFECT-ER intervention and therefore involved in the implementation and action research process. Information sheets were given out by SILs and researchers visited to explain the study further and take consent. All participants provided informed consent (Site A = 80, Site B = 95, Site C = 95). Identifying personal and site features have been changed to maintain anonymity.

### Processes and data collection

Each SIL performed a case note review of ten randomly chosen recently discharged patients with hip fracture and CI, to compare current practice with the XXXX checklist, then creating a score at the start of each PDSA cycle. These results were used to identify areas of strength and improvement. SILs had ward-based action-planning meetings, holding more or less meetings according to their implementation needs, to discuss the results then created and enacted action plans. Change was evaluated using the checklist to find evidence, or not, of service improvement. Action research data comprised: checklist scores from patient case note review, researcher field notes of action-planning meetings, field notes from teleconference meetings with the research team, and SIL generated documents including: their action plans and weekly reflective reports of activities undertaken.

### Analytical approach

We used NPT to analyze and explain the work of agents taking a PDSA approach to implementing and embedding the intervention within different contexts over time. NPT describes four linked sets of mechanisms involved in the normalization of change [10, 11]. The first is ‘coherence’, the ‘sense-making’ work that enables actors to understand new ways of working. The second is ‘cognitive participation’, the work of building relationships so that actors can work together around a change initiative. The third is ‘collective action’ and concerns the interconnected activities of undertaking and sustaining new ways of working. Finally, ‘reflexive monitoring’ relates to the work of appraising the implementation process and informing change in future practices. NPT provides a robust framework for analyzing intervention implementation and is used here to inform comparisons between sites and over time. By considering temporality in the NPT framework, this paper offers new insights into how normalizing work changes over time to deliver sustainable implementation.

### Analytical process

Data from across the sites and PDSA cycles were inductively analyzed by the research team (XX, XX, XX, XX, XX, XX). Following the identification of initial codes, this team collectively developed the coding strategy. Two researchers (XX and XX) used this strategy to code all data to the NPT constructs and PDSA cycles of change. This approach enabled researchers (XX and XX) to apply initial inductive codes to the NPT framework and then move back and forth iteratively between data and theory to identify implementation actions [31]. These were then examined by the whole team and key themes extracted for each NPT construct, site, and cycle, producing a representation of the change process in each site over time. In the next section we present findings for each site before integrating these, examining changes over time and across sites. All Names are pseudonyms to protect confidentiality

## Results

### Findings in Site A

*“…what I learnt from being here 10 years is that people don’t think of ways to improve things day-to-day but just come in and do their job and go home…”* (Elizabeth, SIL Site A, Cycle 1, pre-action-planning meeting with researcher)

As an experienced nurse from an adjoining ward, Elizabeth (SIL, Site A) framed her involvement as an opportunity to get: *“…back to [the] better orthopedic service they had before…”* As illustrated in Table 2, whilst making sense of the intervention checklist, in cycle 1 Elizabeth took time to understand its contents, locating and assessing each item’s legitimacy within Trust policies and national guidelines. Trust policies dictated which items she should implement or not. Organizational support was provided by an ongoing working relationship with Site A process lead Marcus, who she described as “a very influential doctor” on the ward.

**Table 2:** Normalisation process at SITE A.

	Coherence	Cognitive Participation	Collective Action	Reflexive Monitoring
Cycle 1	Locating and meaning making of intervention (in and of itself) in layers of guidance/policies)	Reducing staff burden to engage by preloading required re-organising	Enacting PDSA	Appraising contexts through general experiences and action-planning meeting
	Seeking permissions and exploring boundaries	Gaining of targeted stakeholder support for change implementation	SIL as change agent and explorer of barriers to others being agents for changes	Reconfiguring PDSA processes and checklist
	Understanding target population		Testing: SIL tests checklists contextual compliance	Adaptation: SILs own agenda and checklist moving forward as one
Cycle 2	Realising trust to use own agency / self-accountability in relation to (name removed for peer-review)	Initiating pathfinding and pathfinders for selected checklist items	Enacting PDSA	Information seeking from audit, other disciplines, and evidence sources
	Working out logistics of implementation	Purposeful linking with contextual power brokers	Integrating implementation opportunities into contextual priorities	Lack of support for modifying some practices
				Adapting aspects of ongoing implementation
Cycle 3	Recognition of growth into SIL role	Capitalising and focusing on shared goals and assumptions for shared benefit(s)	Enacting PDSA	Refining and chasing up outstanding implementation tasks
	Brokering knowledge		Visible role modelling implementation in context	SIL reflecting on changes and her role
			Momentum starting work with, and through, others	
Cycle 4	No data coded (Independent SIL)	SIL recognised by context as resource/expert which can be drawn upon	Enacting PDSA	Reflecting on PDSA processes
			Visible role modelling	
			Handing over the momentum and reins	

In cycle 1, Elizabeth’s early sense-making and reflexive monitoring focused on optimising the align of the checklist with the existing ways of working. In particular, she drew on her developed understanding of local practices to re-organise the checklist documentation and its storage prior to the first action planning meeting (see Table 2). At the first action-planning meeting, Elizabeth introduced the intervention, showing how selected changes fitted with Trust policies or national guidance, highlighting low scoring checklist items she wanted to improve. She showed colleagues how these changes would be implemented and what they would need to do to implement them. Importantly, she appealed to colleagues for help:

*“…I can’t implement it on my own*, *I need your help*…*”* (Elizabeth, Site A, Cycle 1, action-planning Meeting).

By developing understanding of the study’s goals and how she contributed to them, Elizabeth became more overtly aware of how the research team related to and relied on the SILs in cycle 2:

*“… the SILs could just fill in the checklist without doing it*, *there is no way for [the research team] to verify scores…*.*she stated she had only just realised this and that we are interested in the process rather than the actual scores…”* (Elizabeth, Site A, Cycle 2, post action-planning meeting with researcher)

In working with the wider research team, Elizabeth started to understand her role as a SIL in different ways: seeing herself more as a ‘change agent’ than as a clinician and relatedly shifting how she engaged in the PDSA process. She focused less on following the checklist and more on engaging the wider clinical workforce in the change initiative (see Table 2).

In Cycle 2 Elizabeth pursued changes to process and practice, strongly guided by local drivers of change agreed by senior management, instead of those driven by the checklist findings. Despite this work, Elizabeth’s records of reflexive monitoring describe how the first time she piloted a change herself, there was a breakdown in communication and therefore a barrier, when a theatre supervisor: *“…had forgotten to cascade information…”* (Elizabeth, Site A, Cycle 2, SIL weekly implementation process report). Elizabeth’s work in cycle 2 centered on understanding change barriers, re-configuring implementation strategies and the roles of stakeholders in these practice changes, to better embed this change (see Table 2).

Change was achieved by making explicit links to organizational knowledge, including the structures and systems already in place such as existing documentation, established actions, trust policies and external influences such as key performance indicators including Best Practice Tariff (BPT), first to herself and then explaining these to staff). This enabled Elizabeth to align to goals shared by the organization and the checklist during Cycle 3, facilitated by pre-existing networks. Coherence and cognitive participation were features of cycle 3, but there was a noticeable shift towards collective action and reflective monitoring. Elizabeth was seen to role-model practices overtly, reflecting and modifying practice changes to improve implementation. Her work additionally energized change already partly embedded. Elizabeth undertook more networking in Cycle 3 to consolidate change involving others, perhaps to anticipate Cycle 4, where change would need to be maintained without her input.

With Elizabeth fully independent in her SIL role, sense-making activities are almost absent from Cycle 4. She understood what the intervention was and knew how it fitted, or not, within her organization. She attempted to change, or not change, items she saw as contextually relevant, and the work needed by her to make sense of her role diminished. Collective action carried the change forward, reducing the emphasis on role-modelling work, whilst the emphasis moved to handing over embedded change to colleagues:

*“…Met with Harriet*, *who has a chief nurse scholarship for one day a week improving care in Theatre Recovery*, *she is interested in how to help…”* (Elizabeth, Site A, Cycle 4, SIL weekly implementation process report).

### Findings in Site B

“*Working together to make realistic and achievable goals*” (Helen, Site B, Cycle 1 Action-planning Meeting).

For Helen, taking the SIL role in Site B enabled her to work at a higher grade (NHS Band 6) within a linked, but different, ward. As shown in Table 3, Helen’s coherence work, in cycle 1, saw her make sense of the intervention’s individual components (checklist, her job description and PDSA process) then as parts of a larger whole. She used reflexive skills to map her current skills to those required by the role: *“…Having some NVQ units to look through for my SIL role was really useful*…*”* (Helen, Site B, Cycle 1, SIL weekly implementation process report). Helen also researched ‘leadership styles’ and how to facilitate open discussions in meetings.

**Table 3:** Normalisation process at SITE B.

	Coherence	Cognitive Participation	Collective Action	Reflexive Monitoring
Cycle 1	Meaning making of intervention elements (checklist, job description, PDSA process)	Increasing enrolment by reducing in required thinking	Enacting PDSA	Information seeking for self-development
	SIL as facilitating collective input	Legitimising, or not, intervention’s cultural stickiness	Being the visible change agent and leader of change	Whole team appraisal of practice and reconfiguration process
	Equipping to develop self	Dynamic networking		Refining PDSA processes
Cycle 2	Targeted information seeking	Increasing enrolment by reducing in required rethinking	Enacting PDSA	Active information seeking from multiple sources
	Facilitator/empowerer	Continuing legitimacy of intervention’s culturally stickiness	Being the visible change agent and leader of change	Reciprocity and full team approach to reviewing and reconfiguration
		Actively seeking the empowerment of networks	Contextual shrewdness: SIL actively seeking out opportunities for social exchange as vehicles for doing	Adaptation: Promotion of checklist and wider ethos
Cycle 3	Recognition of growth with, and within, SIL role	SIL labour as lubricant	Enacting PDSA	Reconfiguring by re-introducing items and introducing new items
	Working, and reworking, active knowledge synthesis	Reorganising community enrolment	Being the visible change agent and leader of change	Embedding changes
		Proliferating contextual authentication	Actively seeking other change agent(s)	Thinking big—changing ethos beyond checklist
Cycle 4	Exploring change legacies	Re-working and re-initiating community activations	Enacting PDSA	Embedding changes
			Being the visible change agent	Continuing momentum
			Handing over changes to others	

Making sense of the PDSA model as part of implementation, Helen undertook extensive work to facilitate the cognitive participation and collective action work of her colleagues in preparing for her first action-planning meeting. She provided Nursing and Midwifery Council (NMC) Continual Professional Development (CPD) documents for attendees to use as evidence of CPD they achieved by attending the meeting;. This work increased the relevance of the action planning activity to nurses and their ongoing registration. Recognizing the multi-disciplinary nature of the intervention, she prioritized specific checklist areas for professions and teams to increase their relevance and reduce work for attendees. Helen encouraged a participatory ethos of input and transparency in her meetings: *“[checklist item] what do we think*? *If [working practices are] not good*, *say…”* (Helen, Site B, Cycle 1, action-planning meeting). The meetings did not produce formal goals; she created these later, then sought management approval before implementing. Changes to documentation and recording practices featured heavily, enabling existing practice to be recognised in the checklist scoring mechanisms.

Helen worked to maintain relevance and buy-in of teams or individuals in cycle 2, reducing coherence-related work and increasing work related to cognitive participation. In cycle 2, Helen used her labor as an opportunity for mutual exchange:

*“…If I support the promotion of MCA [Mental Capacity Act] and support through the CQC [Care Quality Commission] visit I will hopefully get support when I want things for PERFECT-ER …”* (Helen, Site B, Cycle 2, SIL weekly implementation process report).

This reciprocity was unique to this site in featuring strongly across all four cycles (see Table 3).

By tacitly role-modelling practice and documentary changes Helen’s implementation role was key but led to collective action being centered on her own work across cycles 1 and 2. Helen recognized the difficulties with this approach during cycle 3 but continued to use available opportunities to role-model change and to collaborate with others to facilitate implementation. She attempted to embed new practices within the work done by HCAs ensuring that those requiring new skills and competencies became formally recognized in the organization. Producing such recognition within the organization, helped ensure change continued.

Helen brought energy to the implementation, in Cycle 3, by engaging and activating others, for example, arranging and meeting with colleagues engaged in improving care for people living with dementia, to ensure continued investment once her role had stopped [32]. By considering which colleagues she could handover certain changes to, Helen was able to begin to step away from her implementer actions (doing collective action) encouraging others to take these initiatives forward, so securing continued investment and sustaining change. In cycle 4 Helen continued collective action, remaining visible as a ‘doer’, but finalised handover arrangements for changes previously dependent on her efforts.

### Findings in Site C

*“She felt most of the scores were either 100% or 0% and that some of the 0%s were actually being done*, *but not recorded…”* (Karen, Site C, Cycle 1, pre- action planning meeting briefing with researcher).

Karen had not been a ward-based healthcare professional prior to becoming the SIL at Site C thus, she undertook sense-making to orientate herself to her new role (see Table 4). Coherence work was needed to understand the explicit and implicit rules, structures and ethos of the new context. Karen actively framed the intervention, and her role within it which she saw as a way to: “…*highlight all the positive work being done on the ward*… [and that]… *she would become a voice for patients with dementia across the entire hospital…”* (Karen, Site C, Cycle 1, action-planning meeting). As an ‘outsider’ Karen positioned herself as ‘onside’ and thus insider [33]. However, framing the purpose of the checklist, as highlighting “the positive work on the ward” posed a barrier to implementing change when it threatened the ‘virtuousness’ of current practice. She responded to this challenge by asking for changes to the checklist, rather than pushing for changes in practice, to restore the ‘virtue’ of current practice. Karen frequently brought checklist items back to the research team to be altered, illustrating her inability to move beyond coherence as she struggled to internalise the values and benefits of new practices. Instead, she focused on a role as negotiator between Site C and the research team across cycles 1–3.

**Table 4:** Normalisation process at SITE C.

	Coherence	Cognitive Participation	Collective Action	Reflexive Monitoring
Cycle 1	Locating and learning unfamiliar processes and context	Meeting and greeting	Enacting PDSA	Struggling with PDSA processes
	Making value judgements (checklist, practice, patients)	SIL conscription and prescription(s)	SIL making links to stakeholder bodies important in this context	SIL learning through experience
				Reconfiguration focussed on checklist
Cycle 2	Contesting value of checklist, its items and resisting individual changes	Resisting changes to documentation and intervention	Enacting PDSA	Reconfiguration focussed on checklist
	Locating and assessing meaning of potential changes	Pre-arranged actions of SIL & PPL	Promoting	Refining PDSA processes
			SIL making links, and being linked, with others to energise change	
Cycle 3	Recognising growth with, and within, SIL role	Becoming a wider advocate	Enacting PDSA	Monitoring progress with implementation
		Dwindling SIL energy/commitment	Being changed, others who change and being the change	Reflecting on changes as ‘non-ward based’
Cycle 4	Recursive supported SIL	Finding a placed in wider pre-exiting networks	Enacting PDSA	Consolidating outstanding changes
			Handing over identified changes to implementers	Appraising own performance

*“…We scored 100% [on checklist item] initially*, *but [research team] have changed the wording… and we score 0%*… *we will always score 0% and this reflects badly on us…I will take this back* [to the research team]*… as I’m not happy with this as it is not fair…”* (Karen, Site C, Cycle 1, action-planning meeting).

This need to recognize and prioritize current practice rather than trying to modify practice meant that her reconfiguring work focused on trying to change the checklist rather than the related practices.

Implementation can be particularly undermined if those expected to deliver change actually resist it. Karen contested items in particular ways: “*…they* [Karen and Mohamed] *were certain that they did this task*, *just needed to negotiate with* [research team] *to tick the box*…” (Site C, Cycle 2, action-planning meeting). As a small group they could not make sense of the change, because they could not connect with and thus comprehend the wider systems. Karen was supported by the Site’s dementia team, who assisted with sense-making thereby helping to move (who or what?) towards cognitive participation and collective action by being supportive about elements of the checklist contingent to their remit:

*“…[checklist item] was discussed around the table*, *Mohamed [PPL] wanted to know if it was really needed*. *Both dementia workers were very supportive…and felt there were lots of dementia-related issues…*.*that needed recognizing*. *It was agreed that Karen would work with the dementia workers to incorporate a section in the existing policy…”* (Site C, Cycle 1, action-planning meeting)

Over successive cycles, other peers became involved, bringing their wider specialist knowledge assisting with sense-making. This redirected the sense-making, providing new energy, facilitating Karen and Mohamed to move from recursive sense-making into cognitive participation and collective action.

In a further example, during the Cycle 3 action-planning meeting, Mohamed questioned whether particular sets of care practices would be a good thing for patients, even with additional resources. Supporters of specific items (a Dementia Worker and a Senior Physiotherapist) repeatedly advocated their legitimacy and importantly began to suggest solutions. The cognitive participation work in this meeting, enabled Mohamed to recognize the importance of getting: “…*a new female manager*…” on board (Site C, Cycle 2, action-planning meeting). These actions and suggestions for initiating change emerged from others suggesting that even if advocates struggle to see value in proposed changes, peers may show a way to begin to progress changes if they believe it is right for them to be involved. As Karen suggested, “… the new senior physio is really helpful and thinks outside the box…” (Cycle 3, SIL teleconference meeting notes).

Cycle 4 was a winding-down phase at Site C. Karen saw her changes as already handed over and being implemented by others. She stated that she had not been a frontline catalyst for change but had worked to change paperwork with key individuals. She perceived little implementation work was needed in cycle 4 (see Table 4).

## Discussion

### Implementing change: Learning across place and time

We have used NPT constructs to analyse ‘novel’ data and present underlying mechanisms across cases and to provide insights into changes over time.

### Coherence: An intervention legacy

Coherence work at the beginning of implementation featured strongly in these data and appeared necessary when introducing complex change(s), producing a legacy of shared understanding. At Site A, Elizabeth’s prior contextual knowledge enabled her to set up documentary changes ahead of her first action-planning meeting which, in turn, reduced cognitive participation work later required by others. In comparison, at site C, the change agents (Karen and Mohamed) lacked familiarity with the detailed context and engaged more with sense-making work across cycles 1–4. At site B, Helen’s coherence work had similarities to Elizabeth’s, but she undertook more sense-making work in relation to her role to locate its, and thus her, place within the intervention.

Elizabeth’s early coherence work reduced opportunities for colleagues to input here, whereas at site C, Karen’s lack of familiarity with the context, and thus contextual knowledge, indirectly and perhaps paradoxically, encouraged others (physiotherapists and dementia workers) to become involved to assist her sense-making. At site B, Helen’s coherence work in cycles 1 and 2 encouraged colleagues to engage in sense-making work, empowering them to suggest their own change solutions. In these cycles, cognitive participation and collective action focused on enrolling others to invest in the intervention. However, how Helen’s site B colleagues worked together to make-sense of the intervention (communal specification) and the specific implications for their own roles within it (individual specification), resulted in solutions involving Helen directly as the doer. In cycles 3 and 4 this resulted in Helen having to work to reconfigure, re-initiate and rework embedded changes to move the emphasis away from her.

Normalizing processes to embed complex interventions work best when following a clear pathway to a coherent shared goal. Choosing the path, and retaining responsibility for keeping to it, may require less work if those making the choices do not consult widely. In Site A this meant that over time coherence requirements reduced, but the longevity of this goal, and with it the prolonged commitment of others, could prove challenging when others were not collectively invested. Site A’s implementation relied on overt shared goals between the checklist and the organization but, this mechanism of normalization may not engender the continued investment of colleagues.

### Cognitive participation: The costs of widening enrolment

In sites A and B, Elizabeth and Helen held two action-planning meetings per cycle to capture as many colleagues as possible. In site C, Karen held two meetings, however, one of these was a pre-meeting with Mohamed (PPL) where they pre-decided actions. In sites A and C, Elizabeth and Karen used meetings to cascade information about actions they had created. This reduced the requirement for attendees to perform cognitive participation work. Whilst some discussion regarding the feasibility of change occurred, attendees needed to perform little work. In Site B Helen’s work facilitated wider enrolment but at a cost to herself. Helen’s approach to action-planning meetings required more work from her and involvement from attendees. Despite this Helen:

“…*was disappointed no goals had been made, but she was happy everyone was giving ideas*…*She…[will]*…*look at the ideas put forward and run them past the ward manager before implementing them through informal chats*…”

### (Helen, Site B, Cycle 1, post action-planning meeting with researcher)

The legacy of cognitive participation in all sites is clear. At Sites A and C people knew what was required of them when they left the action-planning meetings. Cognitive participation quickly gave way to enacting (or not) changes in their practice. Site B staff made greater investment, engaging in more cognitive participation during action-planning meetings. This had to be translated into action plans and cascaded outside meetings by Helen using a combination of word-of-mouth and visible role-modeling of change solutions. This required more work from Helen and highlights that engaging participants widens the number of perspectives, and so increases the work of generating convergence through collective reflexive monitoring.

### Collective action: Implementing differently

The SILs engaged with challenges that were complex and variable, set against a backdrop of frequent staff change (clinical rotations) and shortages with or without bank or agency staff replacements. Each SIL pursued change differently. Karen focused on documentary change to embed ward-based change and evidenced this from patients’ notes. This reliance on normalizing change through documentation, and having less physical presence in the clinical setting, meant that implementation suffered: *“…they could have jellyfish on their feet for all I know…”* (Karen, Site C, Cycle 3). In other sites being physically present in the ward assisted implementation. Helen role-modelled practice, resulting in collective action relying almost entirely on her actions. Only in cycle 3 did Helen activate others, which then brought new energy to implementation.

Prior to their appointments as SILs change agents came from different work environments, (see Table 1). This influenced how they variously implemented the intervention, having different experiences and networks to draw on. The SILs in sites A and B were ward-based with existing connections to ward-based networks. In site C the SIL was not ward-based prior to commencing the role, meaning connections with formal and informal networks required more work. This affected some ward-based change, which may have been facilitated by established connections with ward networks. Such networks, in every workplace, require individuals to undertake relational or contextual integration work. Reflecting on her lack of integration with ward personnel, Karen stated: “…*if low scoring items on the checklist had been things like bowels* [a ward-based change] *perhaps I would have been more embedded* [in the ward]*…”* (Karen, Site C, Cycle 3, action-planning meeting). Our data suggest generating and facilitating networks, as part of collective action work, enables implementation to become more energized and embedded.

### Reflexive monitoring: Enhancing or inhibiting implementation

Appraisal work may enhance or inhibit implementation across time. When implementation stalled in a site, rotation of new staff offered opportunities for introducing fresh insights and understanding re-energizing impetus. However, frequent movement of staff provided ongoing implementation challenges: “…*she might not follow some of the things we have started…”* (Helen, Site B, Cycle 4, SIL weekly implementation process report). Continuation of implicit informal working practices without tangible consequences were fragile compared to practice supported by management and trust policies with formal consequences.

External influences mitigate the fragility of implementation. For example, practice required by the National Hip Fracture Database (NHFD) BPT has a financial reward based on performance. Practice required by this is not threatened by the need for appraisal work, stimulated by frequent staff changes, because of the tangible nature of its consequences. This is reinforced by the formal collaboratives, put in place by trusts, to ensure such financial reward. In contrast the informal collaborative nature of the PERFECT-ER intervention implementation entailed frequent and ongoing appraisal work constant changes in personnel and a continuing need to undertake repetitive reflexive monitoring work.

Sense making, implementing and normalizing change using a PDSA process implicitly required practitioners to be reflective. Reflection does not fit with ‘delivering more for less’ discourses and as such, its legitimacy and value over more performative/presentational work with instantly tangible/visible outcomes is diminished. Reflection is hidden and therefore ‘invisible work’ [34].

### Wider implications for implementation

Organisational clinical specialties, and the rotational nature of staffing, create barriers that limit the sustainability of change in implementing new ways of working in acute settings [35, 36]. Discourses which emphasise individual accountability before collegiality increase these barriers by discouraging shared sense-making and shared reflection to inform collective action [37, 38]. ‘New professionalism’, in which repertoires of ‘productivity’ or ‘deliver more for less’ condition individualised professional duties, find traction in busy clinical environments which rely on rationalisation to encourage efficiency but discourage coordinated professionally-informed actions [39]. Thus, when trying to deliver sustainable change, (the) formal organisationally-driven collaboratives can enhance implementation-promoting collective actions which align with rationalised healthcare systems. However, this comes at the cost of reducing coherence, cognitive participation, and reflexive monitoring work.

In the acute setting, a short-term ‘ends justifying means’ approach appeals; this is enhanced by material incentives where organisations/institutions reward conformity (for example, NHFD BPT). However, such approaches may ensure more short-term superficial implementation, which may not change individual’s underlying preferences over the long-term. Relying on SILs as implementors requires considering their skills, their experience, and their knowledge of the working setting, all of which will frame their approaches to implementation. To attend to the potential consequences researchers should review both key implementors’ training and their relationships with research and clinical teams.

Restricting individuals’ autonomy may be necessary when an organization’s resources are constrained by hierarchical structures [40], but this can pose a further barrier to change.

If staff autonomy is restricted in the medium to long-term, this affects professional socialisation, a potent social influencing and sense-making mechanism, and so would limit normalisation [41]. The paradox, therefore, for implementing change is to focus on cultures and mind sets relevant to the context, but to also to recognise that bringing people together from different disciplines to be involved in change, will not provide a ready-made uniform culture form to underpin an informal collective’s (coherent) sense-making, or indeed to become a cohesive group. Nor will this readily provide an informal set of working practices required to get things done (collective action). Bringing together different groups and disciplines might therefore lead to more piecemeal and less embedded change. Thus, agents must work to actively cultivate, review, and maintain formal and informal networks, continually reinvesting in these, over time, to sustain engagement [42]. Using NPT to examine change across sites and over time using ‘novel’ data to make has made visible the professional and economic investment needed to maintain change and the context-based facilitative factors. Using such data provided temporality not normally seen in focus group or interview data capturing the ongoing process of implementation.

### Limitations

We sought to implement and refine a new multicompetent intervention in acute hospitals. We relied heavily on SIL generated data without triangulating from other data sources such as other staff in the settings, organizational leads who may have offered alternative perspectives and views and their internal networks. Different change agents had similar disciplinary backgrounds, but their knowledge of ward environments still proved to differ from each other. We have not therefore investigated how other staff disciplines may have influenced implementation.

### Conclusions

We have presented examples of implementation and normalization over time (4 cycles of change) and place (3 acute hospital wards). Despite challenges intrinsic to implementing a novel intervention, each change agent adopted a method of implementing and normalizing change in their ward which could be seen to have contextual fit. Staff collectively activated and operationalized those features that aligned to what motivated in that context. Nonetheless this did not lead to the content and legitimacy of PERFECT-ER being evenly adopted as agents continued to encounter resistance(s) at times and in place. Successful implementation and subsequent normalization were seen to require alignment between perceived values, fit and wider contextual goals. This was important to revisit to ensure sustainability, particularly given the context of constant change which can fragment normalization in both organization and staffing. This meant the role of the change agent (SIL) went beyond successfully introducing the initiative as they continued to sustain the recursive two-way sense-making process required by organizations so as to recognize and respond to staff changes and to engage new energy from incoming staff.

This study reveals how recursive collective sense-making can be. Individuals may not be able to make sense of what they are implementing until different perspectives are brought in. Sense-making moves from individual to collective but also from collective to individual. Fragmented collective insight(s) are brought together by key individuals (in this case the SIL) and then fed back to wider stakeholder groups to foster new collective sense-making and impetus, thereby overcoming impasses in implementation and normalization and working towards sustaining change.

Implementation can be successful, but this is often at some considerable cost to those trying to implement change. Thus implementation–if it happens, is consequently fragile as the present NHS context does not prioritise service improvement and the efforts required for this task thus appears contextually illegitimate.

## List of abbreviations

BPT: Best Practice Tariff
CI: Cognitive Impairment
CPD: Continuing Professional Development
HCA: Healthcare Assistant
NHFD: National Hip Fracture Database
NHS: National Health Service
NMC: Nursing and Midwifery Council
NPT: Normalization Process Theory
PDSA: Plan-Do-Study-Act
SIL: Service Improvement Lead
PL: Process Lead
